# Role of the general practitioner in improving rural healthcare access: a case from Nepal

**DOI:** 10.1186/s12960-018-0287-7

**Published:** 2018-05-10

**Authors:** Bikash Gauchan, Stephen Mehanni, Pawan Agrawal, Mandeep Pathak, Santosh Dhungana

**Affiliations:** 1 Possible, 700 Bluebird Complex, Floor 7, Tripureshwor, Kathmandu Nepal; 2 Division of Hospital Medicine, University of California, San Francisco, San Francisco, CA United States of America

**Keywords:** General practitioner, Healthcare access, Rural, Health worker retention, Education, Quality improvement, Limited resource

## Abstract

**Background:**

There is a global health workforce shortage, which is considered critical in Nepal, a low-income country with a predominantly rural population. General practitioners (GPs) may play a key role improving access to essential health services in rural Nepal, though they are currently underrepresented at the district hospital level. The objective of this paper is to describe how GPs are adding value in rural Nepal by exploring clinical, leadership, and educational roles currently performed in a rural district-level hospital.

**Case presentation:**

We perform a descriptive case study of clinical and non-clinical services offered at Bayalpata Hospital prior to and following the initiation of GP-level services in 2013. Bayalpata is a district-level public hospital managed through a public private partnership by the nonprofit healthcare organization *Possible*. We found that after general practitioners were hired, additional clinical services included continuous emergency obstetric care, major orthopedic surgeries, appendectomy, tubal ligation, and vasectomy. This time period was associated with increased emergency department visits, inpatient admissions, and institutional birth rate in the hospital’s catchment area. Non-clinical contributions included the development of a continuing medical education curriculum and implementation of a series of quality improvement initiatives.

**Conclusions:**

GPs have potential to bring significant value to rural district hospitals in Nepal. Clinical impact may include expanded access to surgical and emergency obstetric services, which would more fully align with local health needs, and could further reduce Nepal’s maternal mortality rate. Task-shifting and structured training programs would be required to increase orthopedic surgery capacity, but this would contribute to meeting local healthcare needs. Non-clinical impact may include supervision of health workers and leadership in continuing medical education and quality improvement initiatives. These changes can lead to improved health worker recruitment and retention in rural posts. Limitations include generalizability of our results to other district hospitals in Nepal and lack of data from control hospitals. This analysis provides an additional perspective on the potential value GPs can add in rural Nepal, through provision of a wide range of clinical and non-clinical services. It supports the expansion of GPs to additional district hospitals in Nepal’s public sector.

## Background

The World Health Organization (WHO) identifies a shortage of more than four million skilled health professionals globally [1]. The global health workforce is unevenly distributed, with many low-income countries considered to be in critical shortage, defined as fewer than 2.28 doctors, nurses, and midwives per 1000 population [1]. The workforce strain is felt most acutely in rural areas, where health worker recruitment and retention continue to be challenging [2, 3]. Balance among health worker types is an important factor when seeking to improve healthcare access [1, 4]. The ideal skill mix among the health workforce varies widely by location and must be informed by local policies, resources, and disease burden [4, 5]. Additionally, task shifting is recognized as an effective tool for improving access to care, especially in low-income countries and rural areas [5]. These global health workforce challenges are felt strongly in Nepal, where mountainous terrain, limited infrastructure, and a critical health worker shortage combine to create significant barriers to healthcare access.

Despite rapid urbanization globally, 81% of people in Nepal live in rural areas as of 2015 [6]. There is a critical health worker shortage in Nepal, with only 0.67 doctors and nurses per 1000 population [7]. To compound this problem, a majority of Nepali health workers are concentrated in the private sector and urban areas [7]. Public district hospitals and primary health centers provide basic emergency and inpatient and outpatient clinical services in rural areas, but these are often inadequate to address healthcare needs.

In the effort to improve access to rural health services, general practitioners (GPs) have been recognized as a key human resource for Nepal [8]. GPs complete a Doctor of Medicine in General Practice (MDGP) residency. The first GP training program in Nepal was established in 1982 at the Institute of Medicine of Tribhuvan University. There are now additional training programs through BP Koirala Institute of Health Sciences, and the National Academy of Medical Sciences, each of which produce 10 to 12 GPs per year.

The GP residency is a three-year structured program in Nepal. The curriculum includes training in medicine, general surgery, obstetrics and gynecology, pediatrics, anesthesia, and emergency medicine. Its goal is to provide graduates with the range of clinical and surgical skills necessary to effectively meet rural healthcare needs, where access to specialists is limited or nonexistent [9]. Analyses of the early effectiveness of these training programs suggest success in producing rural practitioners. A recent study found that 62% of MDGP graduates in Nepal were practicing outside the Kathmandu Valley [10].

In this case study, we will discuss the value added by GPs in a rural district hospital in Nepal that initiated GP-level services in 2013. Extrapolating from lessons learned, we make recommendations for how GPs can be most effectively utilized. We provide an argument for increased investment in GPs within rural Nepal’s public-sector district hospitals.

## Case presentation

### Setting: Bayalpata hospital

Bayalpata Hospital (BH) is a district-level public hospital in Achham District, Nepal. Achham is one of Nepal’s most remote and impoverished districts, with a population of 260 000 [11]. The hospital’s catchment area includes three neighboring districts. It is not uncommon for patients to walk hours to days to reach the facility. District hospitals like BH receive referrals from sub-health posts and primary health centers. They refer on to regional and tertiary care centers. BH lies 14 h by jeep from the nearest major referral center. Since 2009, BH has been managed through a public private partnership (PPP) by *Possible*, a nonprofit health organization. *Possible* strives to innovate within the public sector while filling gaps in service delivery. User fees at BH have been eliminated for all services including surgical, medical, pharmaceutical, and diagnostic.

Recently, the hospital catchment area has seen significant infrastructure development, including new roads, and increased access to electricity and clean water. In addition, the facility has gained increased community trust and awareness in recent years. BH maintains an active community advisory board.

A facility-based electronic health record (EHR) was deployed at BH in 2015 [12]. This now allows for analysis of data trends not possible at most public facilities in Nepal, making BH a suitable target to study.

### Clinical staff

BH’s core clinical staff consists of 30 mid-level providers including paramedics and nursing, along with 6 staff physicians with Bachelor of Medicine and Bachelor of Surgery (MBBS) degrees. MBBS physicians are trained to provide primary and non-surgical health services. Prior to 2013, BH was staffed by MBBS physicians and mid-level providers only. The first GP was hired March 2013, with a goal of expanding clinical services and beginning essential surgical services. There are currently three GPs who oversee the clinical team, coauthors BG, PA, and SD. Additionally, a community health worker program has been implemented and expanded in the catchment area. Community health services included active pregnancy screening, antenatal and postnatal care, contraceptive counseling, and mental health and chronic disease surveillance with counseling. The community health program is linked with BH and local health posts to provide facility referrals when appropriate.

## Methods

We perform a descriptive case study of clinical and non-clinical services offered at BH prior to and following the initiation of GP-level services in 2013.

To assess clinical services, we retrospectively reviewed organizational data on patient and procedural volume. BH has a registry of health services, both paper and electronic. This data is used for government reporting and internal impact assessments. We used routinely generated data on patient volume, stratified by hospital department, to assess trends over time. Data on volume and type of surgical procedures were also obtained from routinely generated reports.

Qualitative data on the orthopedic surgery program were obtained from correspondence with key stakeholders including the orthopedist responsible for training, coauthor MP. Qualitative data on obstetric surgery availability was obtained through correspondence with community health program and hospital leadership. We also cite previously published data on availability of emergency obstetrics care in our catchment area.

We go on to describe non-clinical leadership and programmatic roles played by GPs. The roles discussed align with the defined responsibilities of GPs at BH, which are posted transparently within the organization. Additional information on non-clinical services was obtained through correspondence with hospital leadership. We describe the evolution of a continuing medical education curriculum led by coauthors SD and BG, referencing previously published local data. We review GP involvement in facilitating continuous quality improvement, through coauthors BG, PA, and SD’s firsthand experience. Additionally, we reference previously generated outcome measures from internal quality improvement programs.

## Findings

### Clinical services

After the addition of the first GP at BH, several new clinical and non-clinical services were made available. These are summarized in Table 1 and include cesarean delivery, orthopedic surgeries, daily continuing medical education, and quality improvement initiatives.

**Table 1:** Services at Bayalpata Hospital before and after general practitioners hired in 2013

Before general practitioner availability	Additional services after general practitioner availability
Outpatient	Cesarean delivery
Inpatient	Appendectomy
Emergency	Hernia and hydrocele repair
Medical and surgical abortion	Orthopedic surgery
Non-surgical obstetric service	Tubal ligation and vasectomy
Intrauterine device insertion	Contraceptive implant insertion
Tuberculosis, HIV, leprosy services	Chest tube insertion
Malnutrition service	Daily continuing medical education
X-ray, ultrasound	Morbidity and mortality conferences
Pharmacy	Quality improvement programs
Basic laboratory	Mid-level practitioner certification course
	General practitioner resident rotations

**Fig. 1:**
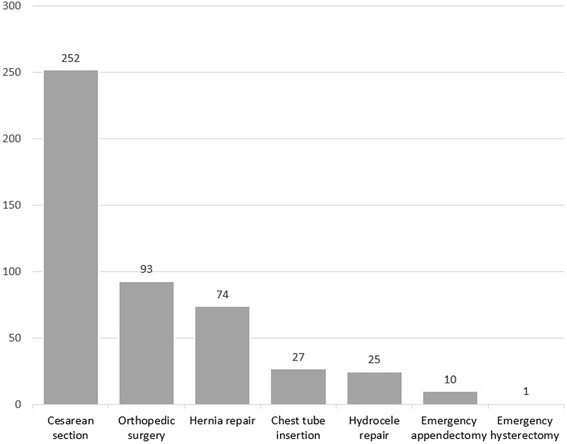
Number of surgical cases performed by general practitioners: 2013 through 2016

Figure 1 shows the number and type of surgical procedures performed after the addition of GPs. From March 2013 through December 2016, GPs performed 252 cesarean sections, 93 orthopedic surgeries, 74 hernia repairs, 27 chest tube insertions, 25 hydrocele repairs, 10 emergency appendectomies, and 1 cesarean hysterectomy independently.

GPs have facilitated the 24-h availability of emergency obstetric care since August 2014. As previously reported, the institutional birth rate in BH’s catchment area increased from 30 to 77% during this time [13]. In a mixed-methods study in the catchment area, quality of health services was identified as a modifying factor for women choosing between institutional or home birth [14]. Importantly, the contribution of GPs accounts for only part of this program’s success. Active surveillance by a community health worker team was critical for identifying early pregnancies, creating a link for health system engagement, and increasing community awareness of services available at BH.

Orthopedic surgeries performed by GPs is also highlighted. There had been an unmet need for orthopedic services at BH, due to the high incidence of traumatic fractures in the catchment area. Because of this, BH leadership and government officials worked to develop regular on-site training for GPs by Kathmandu-based orthopedic surgeons. These trainings have been occurring since 2015, with orthopedic surgeons based at the hospital for periods of 1 week to 6 months. Through this program, GPs have received on-site training to perform major orthopedic surgeries including open reduction and internal fixation, intramedullary nailing, and implant removal. In this task-shifting program, GPs initially perform surgeries under the supervision of an on-site orthopedic surgeon. After the orthopedist has determined the GP has developed the necessary skills and knowledge, the GP begins to perform specified surgeries independently. Ongoing quality assurance is conducted remotely. Pre-operative and post-operative radiographs are shared electronically with the orthopedist to confirm the diagnosis and indication for surgery and to assess immediate post-operative surgical results.

### Patient volume

**Fig. 2:**
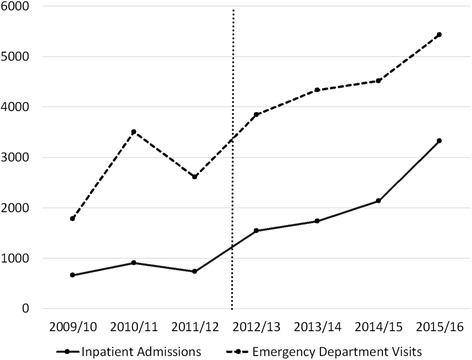
Number of patients in the inpatient and emergency departments at Bayalpata Hospital: 2009 through 2016. Vertical line corresponds to March 2013, when first general practitioner was hired

Figure 2 shows trends in patient volume for the inpatient and emergency departments at BH from 2009 through 2016. There has been a steady increase in patient volume, from 666 hospital admissions in 2009–2010 to 3328 in 2015–2016. A portion of the increased volume is related to the additional clinical services provided by GPs. Prior to the initiation of GP-level services, many of those patients would have been referred elsewhere. Importantly, external variables have contributed to this increase as well. This includes infrastructure development in the area such as roads, electricity and water, a community health worker program, and increased community trust and awareness.

### Medical education and training

GPs have led the development of a continuing medical education (CME) curriculum at BH. The curriculum is delivered by physicians and targeted to mid-level providers and nurses [15, 16]. Four days each week are dedicated to a formal curriculum developed from a local needs assessment. The curriculum focuses on guideline-based management of locally relevant conditions. There are plans to expand this curriculum to Charikot Hospital in Dolakha district, also managed by *Possible.* A weekly morbidity and mortality conference is presented by staff physicians and overseen by GPs, experience from which has been previously described [17]. In addition, GPs facilitate weekly case presentations by mid-level providers. In 2015, in part due to the presence of GPs, BH became a training site for both the government-sponsored mid-level practitioner program [18] and GP resident trainees who rotate for 3 to 6 months.

### Quality improvement initiatives

Prior to the GP arrival to BH, there were no active clinical quality improvement initiatives. In 2015, the first quality improvement program was led by a GP, focusing on hand sanitation in the inpatient department. Outcomes data showed improved adherence to hand-sanitation practices from an initial baseline of 20 to 80% within the first 6 months. Improvements were sustained over time. Another project focused on a government- and hospital-based initiative to screen children under 5 years old for malnutrition. The project resulted in an increase in screening rate of children presenting to the outpatient department from 74 to 92% in the first 3 months. Malnutrition screening results have been maintained at greater than 85% for an additional 12 months to date. More recently, quality improvement initiatives have focused on guideline-based management of chronic obstructive pulmonary disease and gestational diabetes. Outcome metrics are tracked using EHR. The projects are continued for a minimum of 12 months, with ongoing monitoring and program improvements during that time. Additionally, GPs are leading an initiative to standardize clinical care delivery by creating guideline-based protocols for common medical and surgical conditions.

## Discussion

As affirmed at the World Summit on Rural Generalist Medicine, improved access to and quality of care are necessary for health gains in rural communities [19]. Healthcare needs in rural hospitals range from the management of common mild illnesses, to chronic diseases, to emergency medical and surgical conditions. Many people living in rural areas rely exclusively on services provided at their local health facilities. When essential services are not locally available, geography, distance, and cost become prohibitive barriers for referral. The nearest multi-specialist hospitals are 10 to 14 h by jeep from BH. The cost of transport alone is often unaffordable for our patients, the vast majority of whom are classified as “ultra-poor” by the Nepal government. Accepting a referral is a gamble for patients: while cost and inconvenience are certain, a desirable outcome is not guaranteed in emergency situations. Thus, many patients at our facility opt against referral, even when it is unequivocally recommended. Referral centers play an important role in national health systems, but it is important that district hospitals have the capacity to manage the most common medical and surgical conditions. Here we expand on our findings to discuss the value that GPs can bring to rural communities.

GPs can play an important role in rural Nepal by expanding surgical access [20, 21] and access to emergency obstetric services [21–23]. Although Nepal’s success in reducing its maternal mortality rate by more than half in the past 15 years is commendable, a maternal mortality rate of 258 per 100 000 live births is still unacceptably high [24]. A 2011 report found only 6 of 18 (33%) Nepali district hospitals surveyed were able to perform cesarean sections consistently [25]. This is despite evidence that the availability of cesarean delivery can reduce maternal mortality and stillbirth [26]. A study in BH’s catchment area demonstrated institutional capacity and distance from home to be key factors influencing women’s decisions regarding home vs institutional birth [14]. In a single-facility study in rural Nepal, 93% of cesarean sections were performed on an emergency basis [22], supporting the notion that presentations are often late, and timely referrals are infeasible in the remote hills of Nepal.

At BH, a majority of surgeries performed by GPs were cesarean sections (Fig. 1). There have been an average of 90 cesarean deliveries per year at BH since GPs were hired, many performed on an emergency basis. Following the marked improvement in institutional birth rate described above, qualitative data from BH’s catchment area showed that predictors of institutional birth included availability of services and a belief that the hospital was the safest birth location [14]. These data suggest that gains can be made to further reduce maternal mortality by improving access to emergency obstetric services in Nepali district hospitals.

Mirroring our experience, an intervention that deployed GPs to rural district hospitals in Nepal led to increased surgical services, deliveries, availability of emergency obstetric services, and community satisfaction compared to controls [21]. Based on these results, the Nepal government has already taken steps to increasing GP presence at district level hospitals [21].

Orthopedic surgeons, like many specialists, are not available in rural Nepal. Non-orthopedists generally provide much needed basic services, though they lack formal training. GPs do not typically provide orthopedic surgical services. Indeed, orthopedic surgery training is beyond the scope of Nepali GP residencies [27]. However, basic orthopedic services are critical due to the high volume of traumatic fractures. Underscoring this need is the reality that the distance and cost of referral is prohibitive for most of our patients. Thus, significant morbidity or financial hardship result when these surgeries are not performed at the district level. Experience at BH demonstrates that through structured on-site orthopedic training, the range of GP services can be expanded to meet the needs of the communities. Success of a similar task-sharing model has been demonstrated in Malawi [28].

GPs add value in rural posts not only through clinical services but also through educational, programmatic, and leadership roles. Several studies have shown that Nepali medical students are hesitant to work in rural posts after graduation because of lack of professional development opportunities [29, 30] and that MBBS clinicians working in rural posts require additional training [31]. GPs are able to supervise and teach junior doctors and mid-level providers, with potential to expand their scope and improve quality of care. In this way, GPs serve as positive role models with the potential to motivate junior doctors towards rural service [29].

BH’s formalized and structured CME curriculum is an example of where GPs can add value. WHO advocates for continuing education as an evidence-based strategy for improving health worker retention in rural areas [2]. Ideally, continuing education will yield long-term benefits including improvements to medical knowledge, clinical skills, and communication skills. GPs have played an important role developing and improving the CME curriculum, which we hope will lead to improved health worker recruitment and retention. Though this is an outcome we have not yet studied at our site.

Similarly, GPs can provide valuable leadership in quality improvement initiatives. Quality improvement has been championed as a priority for health systems in both high- and low-resource settings [32–34]. Indeed, Nepal is a focus country for the Lancet Global Health Commission on High Quality Health Systems [35]. Despite this, implementation of successful quality improvement programs can be especially difficult in low resource settings, where staffing and project support can be lacking. Quality improvement initiatives often require a robust system to implement, monitor, and evaluate interventions, with iterative cycles of improvement. This can be challenging even in well-resourced settings. In this regard, enhancing human resources has been identified as a priority for improving health systems quality in resource-limited settings [33]. In rural Nepal, GPs—who have more experience with systems-based care than staff physicians or mid-level providers—may be better equipped to lead quality improvement initiatives. GP leadership could underpin a more sustainable model for systems improvement in rural settings.

### Limitations

It is important to note that in isolation, the posting of GPs to Nepal’s district hospitals will be unlikely to have the magnitude of effect described at BH. The hospital is managed by the nonprofit health organization *Possible*, through a PPP with the ministry of health. As such, lines of accountability, salary support, and incentives for innovation differ from other public-sector district hospitals. Under the management of *Possible*, a community health worker program greatly augmented the gains in institutional birth rate. A coordinated orthopedic training program was established in conjunction with the ministry of health to build orthopedic capacity at BH. An integrated EHR helped to facilitate data analysis and quality improvement initiatives at BH and is not yet available in other district hospitals.

Additional limitations include our data set, which is limited to volume of patients and surgical procedures, and internal outcomes of quality improvement initiatives. We do not have the current capacity to report population-level mortality or surgical outcomes data, which limits the conclusions that can be drawn about patient outcomes. We lack long-term data on health worker retention and so are similarly unable to draw firm conclusions of the impact GPs may have. Additionally, we do not have control data from public district hospitals without GPs, which limits the depth of our analysis.

## Conclusion

We provide an additional perspective on the potential value GPs can add to district hospitals in rural Nepal, through the provision of a wide range of clinical and non-clinical services. The robust medical and surgical skillsets GPs bring have potential to align more closely with local healthcare needs. GPs can provide critical supervision, teaching, and leadership to health workers and can facilitate educational and quality improvement initiatives. These services together may ultimately lead to better patient outcomes and improved recruitment and retention of rural health workers. Recognizing the immense challenges that Nepal faces in achieving its commitment to providing equitable, affordable, and accessible care to all Nepalis, we believe that GPs represent a critical component of strengthening Nepal’s health system for the future. This analysis provides additional support to advocate for the placement of GPs in more of Nepal’s 64 public-sector district hospitals. Outcomes and comparative studies from other district hospitals would add generalizability to our analysis. Additional research may also help to elucidate the cofactors necessary for GPs to bring long-term gains to Nepal’s health system.

## Abbreviations

BH: Bayalpata Hospital
CME: Continuing medical education
GP: General practitioner
MDGP: Doctor of Medicine in General Practice
PPP: Public private partnership
